# A Striking Exception to the Chelate Model for Acyclic Diastereocontrol: Efficient Access to a Versatile Propargyl Alcohol for Chemical Synthesis

**DOI:** 10.3390/molecules14125216

**Published:** 2009-12-15

**Authors:** Sami F. Tlais, Ronald J. Clark, Gregory B. Dudley

**Affiliations:** Department of Chemistry and Biochemistry, Florida State University, Tallahassee, FL 32306-4390, USA; E-Mails: stlais@chem.fsu.edu (S-F.T); clark@chem.fsu.edu (R.-J.C)

**Keywords:** pantolactone, synthesis, Felkin–Anh, acyclic diastereocontrol, chiral building block

## Abstract

The four-step, asymmetric synthesis of a chiral propargyl alcohol **1** from (*R*)-pantolactone is described. A key feature of the synthesis is a diastereoselective acetylide addition to a chiral α-alkoxy-aldehyde **7**, in which unusual Felkin selectivity is observed, despite the potential for chelation control. Crystalline propargyl alcohol **1** is valuable for complex molecule synthesis, and is easy to prepare in multi-gram quantities and high diastereomeric purity.

## Introduction

**Figure 1:**
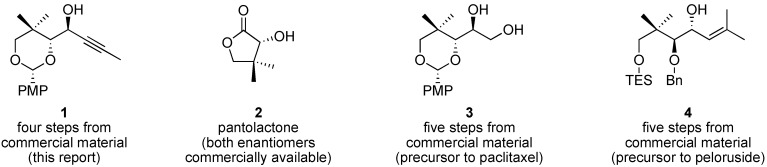
Chiral, functionally rich small molecule building blocks for chemical synthesis.

The chemical synthesis of complex molecules begins with core building blocks [1]. Chiral, functionally rich small molecules serve as starting points in the design of synthetic strategies for front-line molecular targets; knowing which chiral building blocks are readily available is valuable during retrosynthetic analysis. One such chiral building block is the focus of this article; we describe the synthesis, large-scale purification, and X-ray crystallographic analysis of propargyl alcohol **1** (Figure 1) from (*R*)-pantolactone (**2**). In appreciation of the special thematic issue of *Molecules* on Asymmetric Synthesis, a tutorial discussion of steric and stereoelectronic factors that are thought to influence the outcome of a key diastereoselective 1,2-addition reaction is included.

Propargyl alcohol **1** is an ideal starting point for complex molecule synthesis. Both enantiomers of pantolactone (**2**) are commercially available, and **1** offers multiple and orthogonal functional handles for further manipulation. Propargyl alcohol **1** is highly crystalline, which is convenient for purification, storage, and handling of large quantities of material. As an indication of the potential utility of **1** in chemical synthesis, consider that related alcohols **3** and **4** have been converted to the complex natural products paclitaxel (Taxol) [2] and peloruside [3], respectively. Of particular note is that crystalline propargyl alcohol **1** can be prepared in high purity via diastereoselective addition to the corresponding aldehyde (**7**, *vide infra*), despite ambiguity in the Felkin–Anh model and the possibility for chelation control to deliver an alternative diastereomer.

## Results and Discussion

**Scheme 1:**
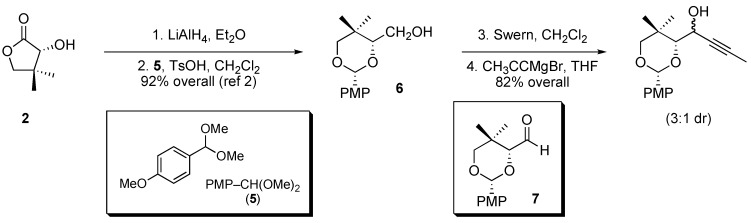
Preparation of propargyl alcohol **1** as a mixture of diastereomers from pantolactone (**2**).

The four-step synthesis of **1** from pantolactone (**2**) is shown in Scheme 1. Reduction of pantolactone with lithium aluminum hydride and selective protection of the resulting triol using *para*-methoxyphenyl (PMP) acetal **5** provides dioxane **6** as reported previously [2]. Primary alcohol **6** is then oxidized to aldehyde **7** under Swern conditions; the unpurified aldehyde is immediately dissolved in anhydrous THF and treated with a solution of propynylmagnesium bromide.

**Figure 2:**
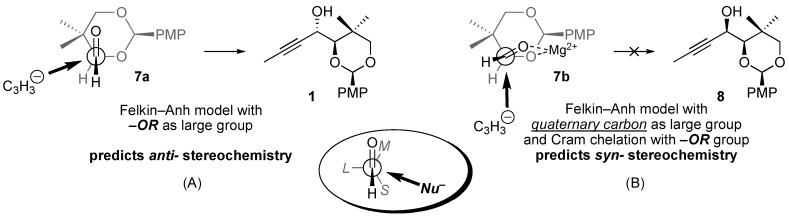
Alternative Felkin–Anh models for predicting/assigning stereochemistry.

Addition of propynyl Grignard to chiral aldehyde **7** provides a 3:1 mixture of two alcohol diastereomers (Scheme 1), the relative stereochemistry of which needed to be assigned. The Felkin–Anh model (Figure 2, inset in oval) does not provide unambiguous guidance in this case, as two groups —namely, the *gem*-dimethyl quaternary center and the electronegative oxygen substituent—could reasonably be designated as the “large” group [4]. Application of the Felkin–Anh model for predicting acyclic diastereoselection in the addition to aldehyde **7** is discussed below.

To apply the Felkin-Anh model (Figure 2), the three substituents on the aldehyde α-carbon (H, OR, and the quaternary carbon bearing the *gem*-dimethyl group) must be designated as “small” (*S*), “medium” (*M*), and “large” (*L*). Typically this designation is made based on the relative sizes of the three substituents (sterics), although there is a stereoelectronic preference for electronegative atoms to act “large” by creating local regions of high electron density that result in negative Coulombic interactions with the incoming nucleophile. Lewis basic substituents typically assume the “medium” position when chelating metal salts are present [5]. Whichever is the relevant substrate conformation, the nucleophile (C_3_H_5_^–^) attacks along the Bürgi–Dunitz trajectory, passing closest to the “small” group and opposite from the “large” group [6].

The reaction path represented in Figure 2B (chelation control, **7b** → **8**) appears quite reasonable, although it proved to be the minor pathway (*vide infra*). Approach of the nucleophile from the side opposite the bulky quaternary center is consistent with steric considerations, and a five-membered ring chelation of magnesium(II) salts may provide a conformational bias in favor of **7b**. The chelation-control model (*i.e.*, Figure 2B) is generally a good predictor of diastereoselectivity in the addition of Grignard reagents to α-alkoxy aldehydes and ketones. For example, allylic alcohol **4** (Figure 1, *vide supra*) is prepared by chelation-controlled hydride addition (reduction) of an α-benzyloxy ketone [3]. 

The reaction path represented in Figure 2A (**7a** → **1**) is favored (in the absence of chelation) on stereoelectronic grounds, as approach of the nucleophile from the side opposite the oxygen substituent minimizes Coulombic interactions between regions of high electron density surrounding the oxygen atom and the electron-rich nucleophile. For the reaction to proceed along this pathway, the five-membered ring chelation of magnesium(II) salts must be disrupted, and the stereoelectronic (Coulombic) shield provided by the electron-rich oxygen substituent must outweigh steric advantages of approaching opposite the bulky quaternary center.

**Figure 3:**
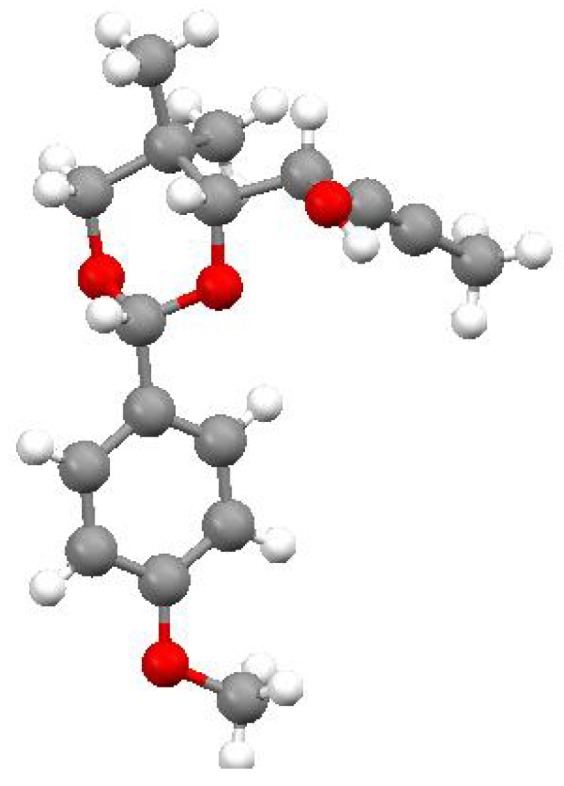
Ball-and-stick representation of propargyl alcohol 1 from X-ray analysis.

**Scheme 2:**
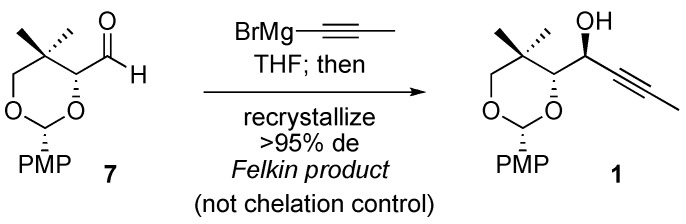
The major diastereomer (1) is highly crystalline.

The major diastereomer produced in the reaction of propynylmagnesium bromide with aldehyde **7** was shown by X-ray crystallography to be **1** (Figure 3), which is the result of Felkin addition in the absence of chelation [7]. This unpredicted result could prove quite useful for chemical synthesis. Aldehyde 7 is available by oxidation of known pantolactone derivative **6** (Scheme 1) [2]. After clean addition of propynylmagnesium bromide, crystallization of the crude product mixture from ether provides monoprotected *anti*-1,2-diol **1** in 40% yield as a single diastereomer (Scheme 2) as measured by ^1^H- NMR spectroscopy [8]. Slow evaporation of a dichloromethane solution of **1** provided crystals suitable for X-ray diffraction analysis (the result of which is shown in Figure 3).

**Figure 4:**
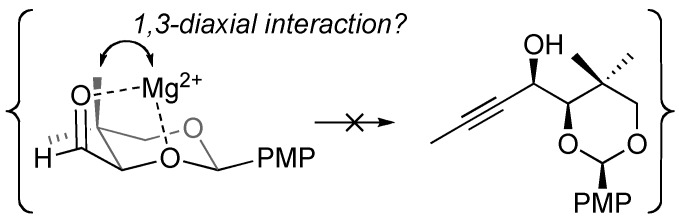
A diaxial interaction that may disrupt chelation in aldehyde 7.

Several factors may be involved in overriding the general tendency for chelation-controlled addition to α-alkoxy aldehydes in this specific case (acetylide addition to aldehyde** 7**). Polar coordinating solvents like THF often erode the diastereoselectivity of chelation-controlled additions, although we observed similar diastereoselectivities when conducting this particular reaction in other solvents. Hyperconjugative interactions (n → σ*) between the acetal oxygens attenuate their Lewis basicity as compared to typical ether linkages, resulting in weaker chelation from acetals, but chelation-controlled addition to α-(alkoxyalkyl)oxy aldehydes is known [9,10] Perhaps the best explanation comes from analysis of an alternative illustration of the potential chelate (Figure 4), which reveals a 1,3-diaxial interaction between the metal cation (with its associated ligands, not shown) and one of the two β-methyl substituents. Such a 1,3-diaxial interaction may disfavor and disrupt chelation.

In the absence of chelation control, selectivity between the competing pathways (cf. Figure 2A and Figure 2B) arises from the balance of steric (favoring *syn*-isomer **8**) and stereoelectronic (favoring *anti*-isomer **1**) factors. Our study focused on the acetylide nucleophile derived from propyne [11]. The steric profile of acetylide nucleophiles is minimal, which minimizes the impact of steric shielding from the large quaternary center [12,13,14]. Meanwhile, the greater ionic character of acetylide organometallic reagents (*sp*-hybridized carbanions) as compared to most *sp^2^*- and *sp^3^*-hybridized carbanions probably translates into greater Coulombic interactions, which repel the electron-rich nucleophile from regions of high electron density. In summary, we attribute the observed stereochemistry to (*a*) a substrate that is ill-suited to chelation of metal salts, and (*b*) a small, electron-rich nucleophile that is more sensitive to stereoelectronic factors than steric factors.

## Experimental

### General

^1^H-NMR and ^13^C-NMR spectra were recorded on a 600 MHz spectrometer and 150 MHz respectively using CDCl_3_ as the deuterated solvent. The chemical shifts (δ) are written in parts per million (ppm) relative to the residual CHCl_3_ peak (7.26 ppm for ^1^H-NMR and 77.0 ppm for ^13^C-NMR). The coupling constants (*J*) were reported in Hertz (Hz). Mass spectra were acquired using electro-spray ionization (ESI+). Melting points were determined on a Mel-Temp apparatus.

*1-(2-(4-Methoxyphenyl)-5,5-dimethyl-1,3-dioxan-4-yl)but-2-yn-1-ol* (**1**): Oxalyl chloride (64 mmol, 5.5 mL) was added dropwise at –78 °C to a mixture of dry DMSO (85 mmol, 6.0 mL) in methylene chloride (72 mL). After 10 min, a solution of alcohol **6** (42.0 mmol, 10.6 g) in methylene chloride (39 mL) was added dropwise. After 30 min, triethylamine (30 mL) was added to the reaction mixture, which was warmed to ambient temperature over 15 min. The reaction was quenched with H_2_O (100 mL) and extracted with methylene chloride (200 mL × 2). The combined organic layers were washed with saturated aqueous NaHCO_3 _(100 mL). Evaporation of the organic solvent under reduced pressure afforded a pale yellow oil, which was mixed with ether (20 mL) and filtered through a small plug of silica gel with ether (100 mL). Evaporation of the volatile components under reduced pressure afforded 10.2 g of a pale yellow solid (consisting primarily of aldehyde **7**), which was used without further purification in the next step.

The aforementioned crude mixture containing aldehyde **7** was dissolved in anhydrous THF (200 mL) cooled at 0 °C, and treated with a solution of propynylmagnesium bromide (0.5 M, 150 mL, 75 mmol). The reaction mixture was warmed to room temperature over 1.5 h. The reaction was quenched with saturated aqueous NH_4_Cl (200 mL) and then extracted with ethyl acetate (300 mL). Evaporation of the volatile components under reduced pressure afforded a yellow oil, which was subsequently diluted with ether (15 mL). Shortly thereafter (within 5 min), white crystals began to form. The crystalline precipitate was collected by filtration, washed with hexane (150 mL), and dried under reduced pressure to furnish 4.89 g (40% from **6**) of alcohol **1** as a single diastereomer. (mp = 105 °C). ^1^H-NMR (CDCl_3_), δ 0.92 (s, 3H); 1.29 (s, 3H); 1.85 (d, 3H, *J* = 2.0); 2.27 (d, 1H, *J* = 7.7 Hz); 3.58 (d, 1H, *J* = 11.1 Hz); 3.63 (d, 1H, *J* = 11.1 Hz); 3.65 (d, 1H, *J* = 4.4 Hz); 3.80 (s, 3H); 4.46 (m, 1H); 6.89 (apparent t, 1H, *J* = 2.1, 2.8 Hz); 6.90 (apparent t, 1H, *J* = 2.1, 2.8 Hz); 7.44 (apparent t, 1H, *J* = 2.1, 2.8 Hz); 7.46 (apparent t, 1H, *J* = 2.1, 2.8 Hz); ^13^C-NMR (CDCl_3_) δ 3.7, 19.5, 22.0, 32.6, 55.3, 62.9, 77.6, 79.6, 83.1, 87.0, 102.0, 113.6, 113.7, 127.6, 131.0, 160.1; HRMS (ESI^+^): calcd. for C_17_H_22_O_4_Na 313.1416, found 313.1426.

## Conclusions

Alcohol **1** is a versatile chiral building block for chemical synthesis. Based on the observations described in this paper—Alcohol **1** is highly crystalline and accessible by simple Felkin addition of propyne to aldehyde **7**—Monoprotected *anti*-1,2-diol **1** is now readily available for the construction of complex molecular targets.
